# A Standard Dictionary of the English Language

**Published:** 1894-04

**Authors:** 


### BOOK NOTICES.

A Standard Dictionary of the English Language upon original plans designed to give in complete and accurate statement, in the light of the most recent advances in knowledge, and in the readiest form for popular use the meaning, orthography, pronunciation, and etymology of all the words and the idiomatic phrases in the speech and literature of the English-speaking peoples. Prepared by more than two hundred specialists and other scholars under the supervision of Isaac K. Funk, D. D., Editor-in-Chief; Francis A. March, LL. D., L. H. D., Consulting Editor; Daniel S. Gregory, D. D., Managing Editor. Associate Editors: Arthur E. Bostwick, Ph. D.; John Denison Champlin, M. A.; Rossiter Johnson, Ph. D., LL. D. Volume I. New York: Funk

& Wagnalls Company, London and Toronto, 1893. Printed in the United States. Price, half Russia, per volume, $7.50 ; per set, $15.00.

In THE HOMŒOPATHIC PHYSICIAN for October, 1891, appeared an advance notice of the publishing of this great dictionary. The first volume has at last appeared and is now before the Editor.

It is a superb work of over 1,000 pages, embracing the letters A to L inclusive. The pages measure twelve inches by nine, and are arranged with three columns to the page. They are furnished with the Denison patent thumb index, by which one is enabled to turn at once to any letter of the alphabet. As an indication of the magnitude of the work it may be stated that it is now nearly four years since it was begun. There have been engaged in its production two hundred and forty-seven office editors and specialists, also nearly five hundred readers for quotations. Besides, some hundreds of other men and women have rendered effective service in various ways in the defining of words or classes of words. As an indication of a determination to make the Dictionary as complete and authoritative as possible there have already been expended in its preparation nearly five hundred thousand dollars, and by the time the remaining volume is completed this sum will be increased by several hundred thousand. The final cost will be not much less than one million dollars before a single complete copy is ready for the market.

It contains more terms than any dictionary before the public.

The full number of words and terms in the several dictionaries for the entire alphabet is as follows: *Johnson*, 45,000; *Stormonth*, 50,000; *Worcester*, 105,000; *Webster* (International), 125,000; *Century* (six volumes, complete), 225,000; STANDARD, nearly **300,000.**

If a word has two or more meanings, the most common meaning has been given first. That is, preference has been given to the "order of usage" over the historical order. The aim has been to remove everything that stands between the vocabulary word and the meaning most generally sought after by the average reader, and, in this way, to enable him to get the information desired with ease and certainty. The obsolescent and obsolete meanings and the etymology are given last.

The Scientific Alphabet, prepared and promulgated by the American Philological Association, and adopted by the American Spelling Reform Association, has been used in giving the pronunciation of words. The powers of the letters are similar to those used in the orthography of the United States Board on Geographic Names and the Royal Geographical Society of England, and in the pronunciation of the great Historical Dictionary of the Philological Society of England (Dr. Murray's). Almost all the prominent linguistic scholars of the two countries are members of one or more of the philological bodies above mentioned, the American and the English. The Scientific Alphabet is a very valuable aid to exact pronunciation. This branch of the work has been under the editorial charge of Professor Francis A. March, of Lafayette College, who is recognized in Europe and America as one of the most eminent of living philologists.

Disputed spellings and pronuncitions have been referred, under the direction of Professor March, to an Advisory Committee of fifty philologists in American, English, Canadian, Australian, and East Indian universit es, and representative professional writers and speakers of English. By a simple system, the forms preferred by each member of the Committee, and those preferred by the leading dictionaries are given in the Appendix to the Dictionary. The preference of this committee is advisory to Dr. March; it is not mandatory.

This great work is not a revision of an old dictionary, but really a new work, intended to take its place as the leading dictionary in the United States; and so complete is its character, and so carefully has the work been done that it is most certain to become, wherever the English language is spoken or studied, one of the few standards to which philologists will turn. Of co irse, there are many who will somewhat object to what is known as the American style of spelling, but no such objection can be raised against *this* dictionary, because disputed spellings and pronunciations have been referred, as already stated, to an advisory committee of fifty philologists in American, English, Canadian, Australian, and East Indian universities, and representative professional writers and speakers of English. The differences of pronunciation are shown in the appendix of the book, and further, any disputed spellings and pronunciations will be found given by each member of that committee. Many words specially relating to chemistry will be found in a changed form, the nature of which may be gathered from the word sulphur, which is spelt sulfur, a style adopted also for all of its derivatives, as sulfate, sulfide, sulfuric, etc. The final "e" is dropped in such words as sulphide, sulphite, etc. These changes have been adopted principally at the desire of the chemistry section of the American Association for the Advancement of Science. Such forms as center and centre are bracketed, and other simple devices are used to suggest a new spelling or to show how the old has been departed from; for the work has not been prepared for American use only, but is designed to be equally useful and acceptable wherever English is spoken.

A point of great importance is the grouping of derived words around their primitives. This was a feature introduced by the Century, but it has been immensely enlarged upon in the Standard, and a great many compound words that needed only the briefest definition have been brought under the chief word from which they were derived and defined by a single phrase suited to each case. This plan saves space and time. Take the word "box," and after the definition of the way in which it is employed in different senses, there is half a column given to the phrases in which it is the leading compound. These phrases are printed in broad-faced type, and practically exhaust the compounds which the word 'box" enters into. This is convenient for the one who consults the dictionary, and it saves important space which is needed for the new words which science and various industries are constantly adding to the language. This comprehensiveness of definition, in which a great number of the phrase words and technical terms were explained, gave the Century one of its most distinctive merits, and the makers of the Standard have been quick to utilize it in their own work. It is of immense service to the student to have the stem word and its compounds with other words grouped where they can be read immediately, and hundreds of times in the Standard long lists of scientific terms are brought together under the heading of the root which enters uniformly into all their compounds. In developing this new feature in the grouping of words the Standard has reached a higher degree of perfection than seemed to be possible, and it has often gone far beyond the Century in its grouping of phrases around the central word under definition.

The Dictionary is amply illustrated; for not only does the text abound with excellent cuts, but there are numerous whole-page plates, with drawings and reproductions of almost every typical thing or class of objects. A special meed of praise is due to the high-class colored plates, than which nothing could be finer; they are the best illustrations of the kind we have yet seen, and they reflect the greatest credit on all concerned in their production.

The enterprising firm who have created this magnificent work deserve the highest praise, their names circulated wherever the English language is spoken, and their Dictionary to be purchased by every literary and educated person in the civilized world.

We would suggest to them that they should devise a dictionary holder capable of accommodating both volumes, and of a style to match their magnificent book.

